# Predictive Performance of Raman Spectroscopy in Osteoarthritis: A Systematic Review

**DOI:** 10.1007/s10916-025-02304-x

**Published:** 2025-11-21

**Authors:** Monira Yesmean, Bijay Ratna Shakya, Minna Mannerkorpi, Simo Saarakkala, Miia Jansson

**Affiliations:** 1 Research Unit of Health Sciences and Technology, Faculty of Medicine, University of Oulu, Oulu, Finland; 2 Biocenter Oulu, University of Oulu, Oulu, Finland; 3 Department of Diagnostic Radiology, Oulu University Hospital, Oulu, Finland; 4 RMIT University, Melbourne, VIC Australia

**Keywords:** Osteoarthritis, Predictive accuracy, Raman spectroscopy, Risk of bias

## Abstract

Early diagnosis of osteoarthritis (OA) remains a critical unmet need due to the lack of reliable detection methods. Detecting OA at an early stage provides a valuable clinical window for implementing effective intervention strategies. Raman spectroscopy (RS) holds promise for improving predictive accuracy in detecting osteoarthritic changes at the molecular level, monitoring disease progression, and assessing severity. This study aimed to systematically evaluate the predictive performance of RS in OA assessment in human samples, thereby highlighting current advancements in the field. The search included PubMed/Medline, Scopus, Web of Science, and IEEE for studies published up to July 31, 2024. Two authors individually screened the studies using Covidence software, and data extraction was based on predefined criteria. The Prediction Model Risk of Bias Assessment Tool was employed to evaluate the bias and applicability of the included studies. Ten studies met the inclusion criteria. Near-infrared excited RS was the most used RS technique. All included studies reported predictive accuracy ranging from 73% to 100% in preclinical settings for OA assessment. Although all studies performed internal validation, most had a high risk of bias and none reported external validation, which limits the generalizability of their findings. These findings underscore both the potential and current limitations of RS in OA assessment. Future research should prioritize larger sample sizes, external validation, and standardized RS protocols to improve reproducibility across diverse clinical settings.

**Supplementary Information:**

The online version contains supplementary material available at 10.1007/s10916-025-02304-x.

## Introduction

Currently, over 595 million people worldwide suffer from osteoarthritis (OA), accounting 7% of the global population [1, 2]. The prevalence of OA is expected to rise significantly, driven by global population growth, increasing obesity rates, and aging demographics [3–5]. OA is a prevalent musculoskeletal disorder affecting multiple joint components, including articular cartilage, synovial fluid, synovial membrane, subchondral bone, meniscus and ligaments at both the macroscopic and molecular levels [3, 6, 7]. The etiology of OA is heterogenous, with recognized modifiable and non-modifiable risk factors including aging, obesity, female gender, hormonal profiles, genetic predisposition, race, acute injuries, chronic overload, and metabolic syndrome [3, 6–8]. OA primarily affects articular cartilage, leading to its degradation through an inflammatory process that breaks down key organic matrix components like collagen and proteoglycans. This degradation not only roughens the cartilage surface but also contributes to chondrocyte death, exacerbating joint deterioration, thickening the subchondral bone, and eventually causing severe joint pain and mobility loss [3, 6, 7].

Medical history, physical examination, and clinical findings remain fundamental for diagnosing OA. Clinical examination is often supplemented by conventional radiography, and in some cases magnetic resonance imaging (MRI), for confirming the diagnosis and assessing disease progression [1, 9, 10]. Radiographic features of OA include joint space narrowing, osteophytes, and subchondral sclerosis, typically summarized by the Kellgren-Lawrence (K/L) grading system. However, the K/L grade is influenced by the evaluator subjectivity and provides an overall joint score rather than a compartment-specific assessment. This can result in discrepancies in OA staging and misinterpretation of the disease’s true extent, particularly in early OA confined to a single compartment [11]. MRI offers superior contrast in soft tissues without the risk of radiation and provides more detailed imaging of cartilage, subchondral bone and meniscal pathology [10]. However, MRI is costly, time-consuming, and has limited availability [12, 13]. Joint fluid analysis, currently the only available laboratory test, is invasive, unsuitable for routine diagnostics, [14] and of limited diagnostic value in OA [15]. A major limitation of these diagnostic techniques is their inability to detect early degenerative changes in cartilage or cellular-level lesions associated with early-stage OA [16–18]. Consequently, these methods often necessitate secondary diagnostic approaches and remain both time-consuming and costly. This underscores the growing need for a diagnostic tool that is objective and sensitive enough to detect early degenerative changes at the molecular level, ideally before irreversible structural damage occurs, enabling timely OA diagnosis and improved clinical outcomes.

Raman spectroscopy (RS) has emerged as a promising alternative to address these gaps. Unlike radiography and MRI, which primarily capture structural changes, RS provides biochemical and molecular information by measuring the inelastic scattering (Raman scattering) of monochromatic light within sample molecules. When a laser illuminates the sample, the energy difference between incident and scattered photons (Raman shift) produces a spectrum that serves as a unique molecular fingerprint, enabling subcellular analysis. Thus, RS has the potential to reveal subtle biochemical alterations that precede the morphological damage detected by radiography or MRI [19–21]. Although challenges such as weak signal intensity and potential interference from fluorescence may limit its effectiveness in some applications [20, 22, 23], ongoing advances in instrumentation, signal pre-processing techniques, and machine learning (ML)-based classifiers can help improve its sensitivity and predictive performance. RS has been utilized in pre-clinical studies of OA for decades [24, 25]. Recently, considerable research has focused on using RS in OA assessment, aiming to improve the prediction and monitoring of joint pathology, evaluating cartilage properties, and disease progression [13, 23, 26, 27].

While existing reviews provide a theoretical foundation and a general overview of vibrational spectroscopy and RS application in OA research [22, 28–30], this review specifically aimed to evaluate the predictive performance of RS in OA assessment against various reference standards in human samples. This will help to evaluate the potential of RS in assessing OA severity, including tissue characterization and OA grading, thereby highlighting current advancements in the field.

## Materials and Methods

### Study Design

This systematic review was performed following the established guidelines for systematic reviews and meta-analysis of prediction model performance [31] and reported according to the Preferred Reporting Items for a Systematic Review and Meta-Analysis (PRISMA) of Diagnostic Test Accuracy Studies statement [32]. The review protocol was registered in the International Prospective Register of Systematic Reviews (PROSPERO; CRD42024559069). The registered PROSPERO protocol, titled *“The accuracy of Raman spectroscopy in the diagnosis of osteoarthritis: A systematic review and meta-analysis*,*”* was updated to reflect the current title and scope of the study. A meta-analysis was not performed due to heterogeneity in sample types, ML algorithms, RS techniques, reference standards, and outcome measures, as well as the limited number of eligible studies.

### Data Sources and Search Strategy

A systematic search was conducted in PubMed/Medline, Scopus, Web of Science, and IEEE up to July 31, 2024, without restrictions on the start date. The search terms included: [“Raman Spectro*” OR “Raman Spectrum” OR “Raman Scattering” OR “Raman Optical Activity Spectroscopy” OR “Spectrum Analysis, Raman” AND “Degenerative Arthritis” OR “wear-and-tear Arthritis” OR “Degenerative joint disease*” OR “chronic non-inflammatory arthritis” OR “osteoarthritis”]. Information specialists assisted in executing this process. No restrictions were applied to language or study type in the initial literature search. The bibliographies of the retrieved articles and relevant unpublished clinical trials were also reviewed to identify additional findings. The search strategy was structured around RS and OA, based on the rationale that studies applying ML within this context would be inherently captured by this broader approach. Citation chaining was further employed to ensure inclusion of all relevant studies combining RS with ML. The comprehensive search strategy is outlined in Table 1.

**Table 1:** Boolean search terms used in databases

Databases	Search terms	Number of references
Scopus (31.7.2024)	(TITLE-ABS-KEY (“Raman Spectro*” OR “Raman Spectrum” OR “Raman Optical Activity Spectroscopy” OR “Raman Scattering” OR “Spectrum Analysis, Raman”) AND TITLE-ABS-KEY (” Degenerative Arthritis” OR “wear-and-tear Arthritis” OR “Degenerative joint disease*” OR “chronic non inflammatory arthritis” OR osteoarthritis))	163
PubMed (31.7.2024)	((“Spectrum Analysis, Raman“[Mesh]) OR (“Raman Spectro*“[Title/Abstract] OR “Raman Spectrum“[Title/Abstract] OR “Raman Optical Activity Spectroscopy“[Title/Abstract] OR “Raman Scattering“[Title/Abstract])) AND ((“Osteoarthritis“[Mesh]) OR (“Degenerative Arthritis“[Title/Abstract] OR “wear-and-tear Arthritis“[Title/Abstract] OR “Degenerative joint disease*“[Title/Abstract] OR “chronic non inflammatory arthritis“[Title/Abstract] OR Osteoarthritis[Title/Abstract]))	86
Web of Science (31.7.2024)	((ALL=(“Raman Spectro*” OR “Raman Spectrum” OR “Raman Optical Activity Spectroscopy” OR “Raman Scattering” OR “Spectrum Analysis, Raman” AND “Degenerative Arthritis” OR “wear-and-tear arthritis” OR “Degenerative joint disease*” OR “chronic non inflammatory arthritis” OR osteoarthritis)) AND TS=(“Raman Spectro*” OR “Raman Spectrum” OR “Raman Optical Activity Spectroscopy” OR “Raman Scattering” OR “Spectrum Analysis, Raman”)) AND TS=(“Degenerative Arthritis” OR “wear-and-tear arthritis” OR “Degenerative joint disease*” OR “chronic non inflammatory arthritis” OR osteoarthritis)	121
IEEE (31.7.2024)	(“Full Text & Metadata”:Raman) AND (“Full Text & Metadata”:Spectro* OR “Full Text & Metadata”:Spectrum OR “Full Text & Metadata”:Scattering) AND (“Full Text & Metadata”:arthritis OR “Full Text & Metadata”:osteoarthritis)	98

* Truncation of a search word

### Eligibility Criteria

The first and second authors individually screened the titles and abstracts of studies using the automation tool, Covidence software (https://www.covidence.org/). They assessed the studies’ eligibility based on predefined criteria. The full texts of eligible studies were then reviewed. When the authors had differing opinions, these were resolved by reaching a consensus through discussion. Our selection criteria included only peer-reviewed journal articles that utilize RS to assess OA in human samples. If a study contained both human and animal samples, only the human samples were considered. Studies were selected according to the following PICOTS criteria:

- Population: Adult patients with OA.
- Intervention: RS.
- Comparator: Reference standard.
- Outcome(s): Predictive performance metrics (e.g., accuracy, sensitivity, specificity).
- Timing: Prediction models to be used prior to and at the moment of assessment.
- Setting: Ex vivo, in vivo, in vitro settings.

Studies were excluded for:

- The absence of reported predictive performance metrics.
- Irrelevant article types such as reviews, duplicate reports, letters, editorials, and case reports.

### Study Selection

A total of 468 articles were retrieved from four search databases. Additional manual searching was conducted to identify further relevant articles. This included two articles found through a web search and one identified via citation searching. After removing duplicates, 285 articles remained for screening. The first and second authors independently screened the titles and abstracts, narrowing the selection to 13 articles that met the specified eligibility criteria for full-text reviews. Three articles were excluded: one was a conference paper, another used a duplicate dataset, and the third did not report any predictive performance metrics. Ultimately, ten articles were included in this review.

### Data Extraction

Data from all eligible studies were extracted following upon the checklist from the Critical Appraisal and Data Extraction for Systematic Reviews of Prediction Modelling Studies [33]. This checklist covers various parameters, including data sources, sample details, index tests, predictors, reference standards, sample size, handling of missing data, model development process, model performance metrics (accuracy, sensitivity, specificity), model evaluation, and overall results. The first author performed the initial data extraction, which was then cross-verified with the original articles by the third author. Any differences in opinion were resolved through discussion between the first and third authors until a consensus was achieved.

### Literature Quality Assessment

The first and second authors individually evaluated the quality of all included studies with the Prediction Model Risk of Bias Assessment Tool (PROBAST) [34]. Disagreements were resolved through discussion with the third author. This quality assessment was ranked into four major categories: participants, predictors, analysis, and outcomes. The risk of bias (ROB) was labeled as low, high and unclear. When all categories were marked ‘low’ ROB, models without external validation were still deemed ‘high’ ROB unless they’re based on a very large sample set and included some form of internal validation [35]. In line with the Cochrane [36], ROB assessments were used to interpret the results of the included studies, and the associated limitations were explicitly addressed in the synthesis and interpretation. All eligible studies were retained and synthesized narratively, with interpretations made cautiously to account for potential bias [31, 36]. Sensitivity analyses were not conducted because no meta-analysis was undertaken [36].

## Results

### Study Selection

**Fig. 1:**
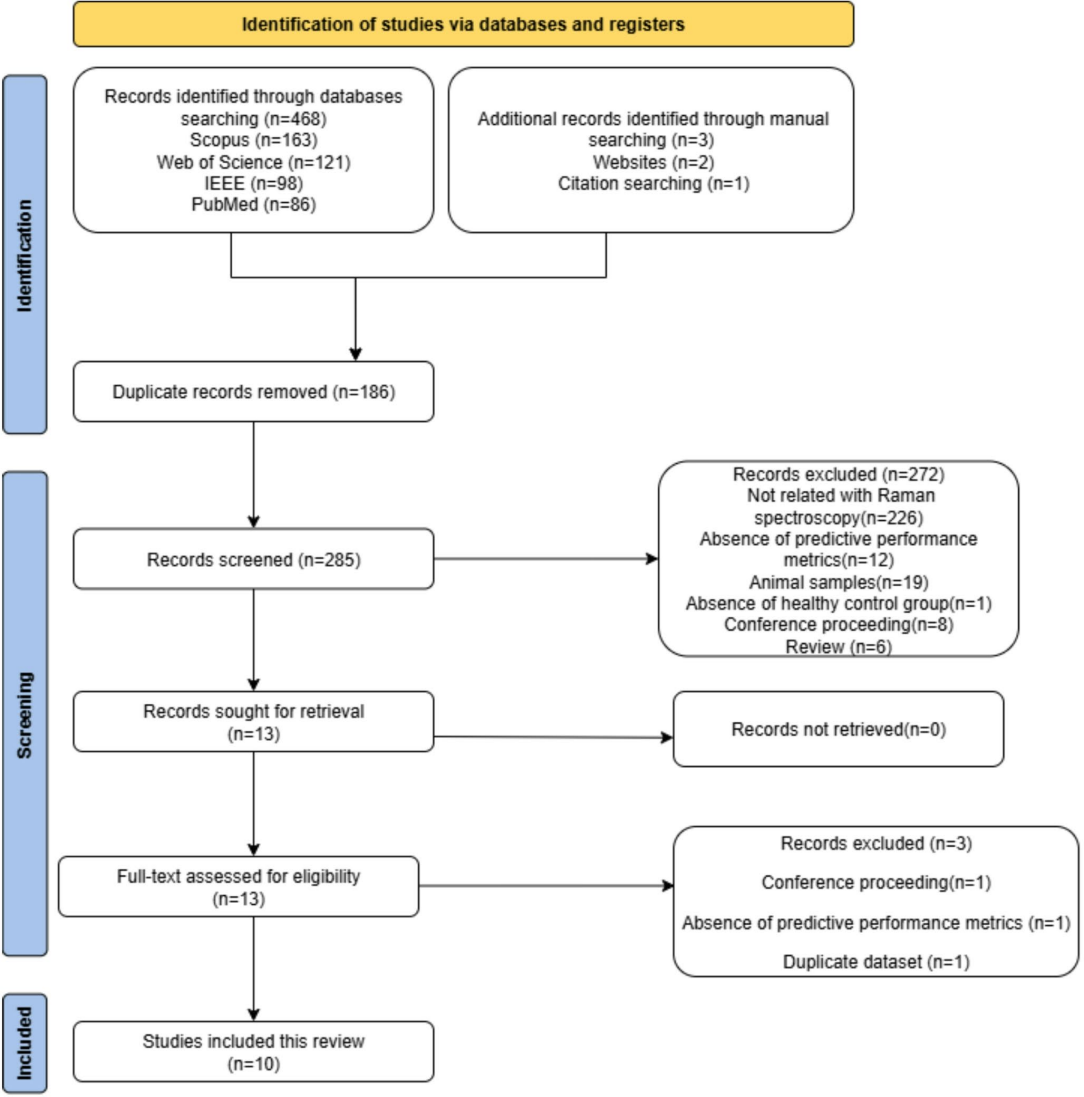
PRISMA flowchart of the study selection procedures

The details of screening, study identification, inclusion, and exclusion in this review are depicted in the PRISMA flowchart (Fig. 1).

### Basic Information of Included Studies

The basic information for the ten studies included in this review is detailed in Table 2. Out of ten, nine studies adopted an ex vivo study design [37–45], while only one study Richardson et al. [46] employed an in vitro study design. The samples in the included studies were predominantly derived from knee joints (*n* = 7), followed by hip joints (*n* = 3). The studies varied in sample types, which included articular cartilage (*n* = 6), synovial fluid (*n* = 2), meniscus (*n* = 1), and osteochondral samples (*n* = 1).

**Table 2:** Data extraction of predictive performance of RS in OA assessment

Study	Sample source (Sample type)	Participants (Sample size)	Reference standard	Index tests	Missing data	Model type	Model evaluation	Model performance metrics	Overall results
Bocsa et al. 2019	Knee (SF)	23 (23)	Clinical criteria	RRS & SERS	NR	PCA-LDA	LOOCV	ACC, SEN, SPE	For Knee OA grading: RRS & PCA-LDA: ACC 74%, SEN 67%, SPE 82%; SERS & PCA-LDA: ACC 91%, SEN 92%, SPE 91%; concatenated (RRS & SERS) with PCA-LDA: ACC 100%, SEN 100%, SPE 100%
Cardinali et al. 2023	Hip (AC)	5 (10)	Clinical criteria & K/L grading	BRam’s	NR	PCA-LDA	IV	AUC, ACC, SEN, SPE	BRam’s & PCA-LDA: ACC for healthy, doubtful, minimal, moderate, severe OA grading: 74%, 60%, 92%, 96%, 100%; SEN for healthy, doubtful, minimal, moderate, severe OA grading: 74%, 98%, 99%, 100%, 100%; SPE for healthy, doubtful, minimal, moderate, severe OA grading: 91%, 99%, 100%, 100%, 100%
Cook et al. 2024	Hip (AC)	27 (81)	Histopathological grading	NIR- RS & NIR- SWIR	NR	PCA-LDA & SVM	k-fold CV, LOOCV	ACC, SEN, SPE	NIR-RS & SVM models classified osteoarthritic degenerated tissue: ACC 78%, SEN 77%, SPE 79%; concatenated (NIR-RS & NIR-SWIR) with SVM: ACC 85%, SEN 77%, SPE 93%; NIR-RS & PCA-LDA models classified osteoarthritic degenerated tissue: ACC 78%, SEN 77%, SPE 79%; concatenated (NIR-RS & NIR-SWIR) with PCA-LDA: ACC 100%, SEN 100%, SPE 100%
Crisford et al. 2024	Hip (AC)	64 (256)	Histopathological grading	NIR- RS	NR	PCA-LDA	k- fold CV	ACC	NIR-RS & PCA-LDA classified superficial and deep layers in diseased cartilage: ACC 88% and 84%, respectively; NIR-RS & PCA-LDA classified superficial and deep layers in healthy cartilage: ACC 97% and 93%, respectively
Esmonde-White et al. 2009	Knee (SF)	40 (37)	K/L grading	NIR- RS	Excluded	K-means	IV	ACC, SEN, SPE	NIR-RS & K-means for Knee OA diagnosis: ACC 73%, SEN 74%, SPE 71%
Kumar et al. 2015(a)	knee (AC)	3 (12)	Histopathological grading	CRS	NR	PCA	LOOCV	ACC, SEN, SPE	CRS & PCA for osteoarthritic cartilage grading: ACC for grade I, II, III: 85%, 88%, 96%; SEN for grade I, II, III: 81%, 85%, 89%; SPE for grade I, II, III: 87%, 90%, 100%
Kumar et al. 2015(b)	knee (AC)	5 (15)	Histopathological grading	CRS	NR	PCA	LOOCV	ACC, SEN, SPE	CRS & PCA for osteoarthritic chondrocyte grading: ACC for grade I, II, III: 99%, 92%, 93%; SEN for grade I, II, III: 99%, 83%, 98%; SPE for grade I, II, III: 100%, 98%, 91%
Prokopi et al. 2021	knee(menisci)	27 (27)	Histopathological grading	PRS	NR	NR	IV	SEN, SPE	PRS for non-degenerated & degenerated meniscus grading: SEN 83%, SPE 91%
Richardson et al. 2015	Knee (AC)	NR (8)	Histopathological grading	RS	NR	PCA-LDA	LOOCV	SEN, SPE	RS &PCA-LDA for healthy and osteoarthritic cartilage: SEN 99%, SPE 93%
Tafintseva et al. 2025	Knee(osteochondral)	18 (272)	Histopathological grading	NIR- RS, MIR, NIR	NR	PCA-SVM, PLS-DA, RF	LOOCV	ACC, SEN, SPE	NIR-RS & PCA-SVM predicted cartilage degradation: ACC 78%, SEN 75%, SPE 80%; concatenated (NIR-RS, MIR & NIR) with PCA-SVM predicted cartilage degradation: ACC 81%, SEN 79%, SPE 83%

*AC* Articular cartilage, *ACC* Accuracy, *AUC* Area under the curve, *BRamS* Brillouin and Raman micro-spectroscopy, *CRS* Confocal Raman spectroscopy, *CV* Cross-validation, *IV* Internal validation, *K/L* Kellgren-Lawrence, *LDA* Linear discriminant analysis, *LOOCV* Leave-one-out cross-validation, *MIR* Mid-infrared, *ML* Machine learning, *NIR* Near-infrared, *NIR-RS* Near-infrared Raman scattering spectroscopy, *NR* Not reported, *OA* Osteoarthritis, *PCA* Principal component analysis, *PLS-DA* Partial least squares discriminant analysis, *PRS* Polarized Raman spectroscopy, *RF* Random forest, *RS* Raman spectroscopy, *RRS* Resonant Raman spectroscopy, *SEN* Sensitivity, *SERS* Surface-enhanced Raman scattering, *SF* Synovial fluid, *SPE* Specificity, *SVM* Support vector machine, *SWIR* Short-wave infrared

### Types of RS

Four of the included studies utilized near-infrared excited RS (NIR-RS) [39, 40, 43, 45], two studies employed confocal RS (CRS) [37, 38], one used Brillouin and Raman micro-spectroscopy (BRamS) [41], one applied polarized RS (PRS) [42], one used conventional RS [46], and another utilized resonance RS (RRS) coupled with surface-enhanced Raman scattering (SERS) in OA assessment [44].

### Type of ML Models

ML models used in the studies included the following supervised algorithms: principal component analysis-linear discriminant analysis (PCA-LDA), LDA, and support vector machine (SVM) across six studies [39–41, 44–46]. In addition to PCA-SVM, Tafintseva et al., [45] also employed partial least squares discriminant analysis (PLS-DA) and random forest (RF) algorithms. Unsupervised algorithms, such as K-means clustering and PCA, were utilized in three studies [37, 38, 43], while one study [42] employed univariate analysis. The reference standards for OA assessment varied, with histopathological grading being the most common (*n* = 7), followed by clinical diagnosis (*n* = 2), and X-ray-based K/L grading (*n* = 1). Among the histopathological gradings, three studies [37, 38, 46] utilized International Cartilage Repair Society (ICRS) grading. Two studies [39, 40], employed Mankin scoring, and one study [45], applied Osteoarthritis Research Society International (OARSI) grading.

### Predictive Performance of RS Combined with ML Models in OA Assessment

#### Predictive Performance of RS Combined with ML Models in Osteoarthritic Tissues or Cells Characterization

In all included studies, Raman spectroscopic data were pre-processed, typically using background removal, baseline correction, and normalization, reflecting a consistent methodological approach across studies. NIR-RS was the common modality and PCA-LDA was a frequently used approach for reducing the high dimensionality of Raman spectral data and enhancing class separation. In this approach, PCA was applied first to extract principal components (PCs) that captured variance relevant to group differences, with the optimal number of PCs determined using performance metrics to prevent overfitting. These PCs were then used as inputs for LDA, to maximize separation between predefined OA severity groups. Richardson et al., [46] used RS with PCA-LDA to classify healthy and OA cartilage, selecting the final number of PCs based on the plateau of a class-separation metric. Kumar et al., [37, 38] used CRS spectra with PCA-LDA in both cartilage samples and isolated chondrocytes across ICRS Grades I–III. In cartilage, PC1–PC3 explained 84.00%, 12.00%, and 2.00% of variance with 85% predictive accuracy, while in chondrocytes, the corresponding values were 82.30%, 7.06%, and 3.75% with 92% predictive accuracy. Both studies used leave-one-out cross-validation (LOOCV) with Mahalanobis distance, with the higher accuracy in chondrocytes indicating greater discriminatory power at the cellular level.

Layer-specific performance was evaluated by Cristford et al., [39], who applied PCA-LDA to Raman data from superficial and deep cartilage layers. Using 10 PCs they achieved accuracies of 88% (superficial) and 84% (deep) for diseased tissue and 97% (superficial) and 93% (deep) for healthy tissues. PC1 accounted for 45.9% and 42.5% of variance in superficial and deep layers, respectively. Cardinali et al., [41] combined BRamS with PCA-LDA to classify femoral head cartilage into five OA severity categories, achieving 86% accuracy compared to 84% with LDA alone. PC1 was excluded as it captured focus artefacts. A stratified 75/25 train and test splits were used, with additional tests simulating rapid intraoperative acquisition. Method comparisons and data fusion were examined by Cook et al., [40] who compared the accuracies of PCA-LDA and SVM on Raman spectral data, while Raman scattering and NIR-short wave infrared data were concatenated into a new spectromic data. With 10 PCs, PCA-LDA accuracy increased from 78% (Raman) and 62% (NIR-short wave infrared) to 100% when features were concatenated across spectromic data. Reducing to 5 PCs reduced accuracy for single modalities but retained 85% accuracy for the concatenated spectromics dataset. SVM, optimized via grid search for hyperparameters C and γ, further improved performance to 85% (Raman) and 89% (concatenated), indicating advantages for handling nonlinear, high-dimensional data.

Tafintseva et al., [45] evaluated multiple ML models to classify osteochondral samples as healthy (OARSI grades 0–2) or damaged (grades 2.5–6) using Raman, mid-infrared (MIR), and NIR spectra. They evaluated one block classifier using PLS-DA, RF, and SVM with prior feature reduction via PCA (PCA‑SVM). The highest one‑block classification accuracies was obtained with PCA‑SVM which were 77.5% for MIR and 77.8% for Raman, while the NIR data performed less well at 68.5%. For linear kernel SVM, box constraint (b) was tuned between 0.01 and 3 (optimal: MIR 0.7, NIR 2.12, Raman 0.01) and for PCA-SVM (retaining 38, 36, and 16 PCs for MIR, NIR and Raman) used optimized box constraint (b) values of 0.04, 1.0, and 0.8, respectively. PLS-DA used 6–7 latent variables (LVs) for single-block and 10 LVs for multiblock fusion and RF employed 100 trees. For multi-block (fusion) approach of concatenating all three spectral modalities, PCA-SVM fusion achieved the highest accuracy of 81%, and multiblock PLS-DA attained 79.1%, both significantly better than the single‑block models as confirmed via analysis of variance, highlighting the benefit of multimodal spectral integration for assessing cartilage health.

#### Predictive Performance of RS Combined with ML Models in Knee OA Grading

Esmonde-White et al., [43] employed NIR-RS in combined with K-means clustering to categorize Raman spectral data from synovial fluid, achieving an accuracy of 73%. Instead of curve fitting, the clustering model used pixel intensities related to the 1080 cm^− 1^/1001 cm^− 1^ and amide I ratios as input features. Two predefined groups, corresponding to low-grade (K/L 0 to 1) and high-grade (K/L 2 to 4) OA were used to guide the classification. Although K-means is less powerful than supervised models for direct classification, it offers valuable preliminary insights into data structures by iteratively adjusting cluster centers to minimize within cluster variance, thereby revealing natural groupings. Bocsa et al., [44] developed separate PCA-LDA models for Raman and SERS spectra. Raman spectra alone achieved 74% accuracy, while SERS spectra reached 91% accuracy. When the two spectral types were combined in a simultaneous analysis, the multimodal approach increased classification accuracy to 100%, effectively distinguishing between low-grade and high-grade OA samples. The first 10 PCs were used as input for LDA, and model performance was validated using LOOCV. Prokopi et al., [42] applied PRS with univariate analysis to differentiate low- from high-grade meniscal degeneration, achieving 85% accuracy. However, univariate analysis, being less comprehensive than multivariate models, may benefit from further validation for consistent diagnostic use.

### Spectroscopic Characteristics in Assessing OA Severity

Most studies focused on the fingerprint region (600–1800 cm^− 1^), which is critical for detecting biochemical alterations in joint tissues, especially in articular cartilage and synovial fluid, where Raman bands primarily reflect protein signatures [37–40, 42, 43, 45, 46]. Table 3 summarizes tentative assignments of key Raman spectral predictors from the included studies.

**Table 3:** Outline of tentative assignment of approximate positions of Raman spectral predictors applied for osteoarthritis assessment from the included studies

Raman spectra predictor (cm^− 1^)	Assignment to chemical bonds and molecules
816–817	C-C stretching; protein backbone
853–859	C-C stretching; proline
874–875	Hydroxyproline (C–C stretch)
937–941	C-C stretching; collagen, α-helix
965	PO4^3-^ stretching; phosphated hydroxyapatite
1001–1004	Phenylalanine ring breathing
1060–1064	SO_3_^−^ stretching in sulphated glycosaminoglycans (chondroitin sulfate)
1125–1130	C-C, C-OH, C-N stretching, C-O-C glycosidic linkage
1235	Amide III, random coil
1245	CN stretching of amide bond, Amide III, random coil (disordered)
1260–1272	Amide III, α-helix
1424–1425	COO − glycosaminoglycans
1445–1452	CH_2_, CH_3_ scissoring, NH_2_ deformation of amide bond, collagen and other proteins
1557–1560	Amide II
1606	C = C stretching; phenylalanine, tyrosine
1637–1640	Amide I, collagen secondary structure
1660–1696	C-O stretching; amide I, random coil

The parameters derived from the protein, amino acid, carbonate and hydroxyapatite bands can be used to discriminate between osteoarthritic and healthy samples [29, 47]. Frequently cited peaks in assessing OA severity across all studies include proline (853–859 cm^− 1^), hydroxyproline (874–875 cm^− 1^), phenylalanine (1001–1004 cm^− 1^), proteoglycans (1060–1064 cm^− 1^), amide III (1260–1272 cm^− 1^), organic content (1445–1452 cm^− 1^), amide II (1557–1560 cm^− 1^), and amide I (1660–1670 cm^− 1^) [37–44, 46]. The pronounced spectral difference at these wavenumbers highlights the biochemical variability among the osteoarthritic and healthy samples. Kumar et al. [38] reported reduced intensity in the nucleic acid band (780–794 cm^− 1^), correlating with OA progression. Cardinali et al. [41] observed phosphate (965 cm^− 1^) and carbonate (1064 cm^− 1^) peaks in spectra from weight-bearing zones such as the subchondral plate, indicating mineralized tissue exposure in severe OA (K/L 4). Bocsa et al. [44] noted diminished carotenoid-specific Raman peaks (1003, 1155, 1512 cm^− 1^) in high-grade OA, absent in SERS spectra, likely due to selective enhancement via pre-resonance effects from the 532 nm laser, providing insight into the tissue redox state.

### Assessment of Study Quality

Of the ten studies included, all exhibited a high ROB, specifically within the analysis domain (Table 4). However, all studies showed a low ROB in the other three domains such as participants, predictors, and outcomes, with low risk in applicability as well.

**Table 4:** The risk of bias in the included studies

Author	Risk of bias				Applicability			Risk of bias	Applicability	Overall
	Participants	Predictors	Outcome	Analysis	Participants	Predictors	Outcome			
Bocsa et al. 2019	Low	Low	Low	High	Low	Low	Low	High	Low	High
Cardinali et al. 2023	Low	Low	Low	High	Low	Low	Low	High	Low	High
Cook et al. 2024	Low	Low	Low	High	Low	Low	Low	High	Low	High
Cristford et al. 2024	Low	Low	Low	High	Low	Low	Low	High	Low	High
Esmonde–White et al. 2009	Low	Low	Low	High	Low	Low	Low	High	Low	High
Kumar et al. 2015(a)	Low	Low	Low	High	Low	Low	Low	High	Low	High
Kumar et al. 2015(b)	Low	Low	Low	High	Low	Low	Low	High	Low	High
Prokopi et al. 2021	Low	Low	Low	High	Low	Low	Low	High	Low	High
Richardson et al. 2015	Unclear	Low	Low	High	Low	Low	Low	High	Low	High
Tafintseva et al. 2025	Unclear	Low	Low	High	Low	Low	Low	High	Low	High

## Discussion

The use of RS in OA assessment is a growing area of research, with most studies published between 2019 and 2024. All included studies demonstrated excellent predictive accuracy of RS integrated with ML models for assessing OA, with reported values ranging from 73% to 100% in preclinical settings. However, all studies relied solely on internal datasets and did not test generalizability on external cohorts. Consequently, the risk of bias is high, and their reported performance is likely optimistic. In addition, most studies did not report model calibration or clinically anchored decision thresholds, further limiting the interpretability and clinical translation of their findings. This review also shows that research has primarily focused on characterizing degenerated osteoarthritic cartilage tissues or cells and grading knee OA. The heterogeneous nature of OA, combined with its high prevalence and significant impact on the general population, likely explains the focus on its early detection. Detecting OA at the molecular stage, before structural progression, can be facilitated by RS combined with ML models, thereby improving the understanding and management of the condition.

This review underscores the strong predictive performance of NIR-RS, CRS, and SERS in assessing OA severity. NIR-RS is notably promising for clinical application due to its ability to amplify Raman signals and effectively minimize fluorescence background and phototoxic effects [22]. CRS offers high spatial resolution and intensity, providing detailed biomolecular insights into OA cartilage, leading to more precise diagnostic outcomes [17, 48]. Among these techniques, SERS emerged as particularly promising, as it amplifies the Raman signal up to 10^11^ times, thereby overcoming the low detection probability that typically limits conventional RS [49–51]. This enhancement significantly improves classification accuracy between early and advanced stages of OA.

The integration of RS with ML models has been central to OA assessment, with PCA-LDA, PLS-DA and SVM among the most frequently used algorithms [22, 52]. Integrating ML models into the spectral analysis pipeline has improved the ability to detect subtle and challenging changes within the Raman spectra of biological samples, showing promising results in OA assessment for both preclinical and clinical applications [46, 53–55]. These ML models are capable of effectively analyzing multidimensional RS datasets, identifying complex patterns and relationships in Raman spectra, and classifying biological samples from both healthy and diseased donors [52, 56]. The choice of ML model notably influenced classification performance, with PCA-LDA and SVM combinations generally achieving the highest accuracy in classifying joint tissues of varying OA severity across the included studies. PCA reduces spectral dimensionality by extracting PCs that capture most variance, aiding in pattern detection. When combined with LDA, this approach enhances class separation, particularly when class variances are similar and datasets are relatively small [57]. In contrast, PLS-DA is well-suited for high-dimensional datasets, as it extracts LVs that capture the maximum covariance between predictors and class labels. However, PLS-DA is sensitive to class imbalance and requires tuning of the number of LVs, typically performed through cross-validation. SVMs apply kernel transformations to map input data into higher-dimensional spaces for robust classification, but they require careful hyperparameter tuning to prevent overfitting [57]. Most of the included studies employed LOOCV to optimize model performance, likely due to the relatively small dataset sizes [57]. Future research that combines dimensionality reduction techniques with robust classifiers, such as PCA-SVM, could enhance predictive performance, particularly if external validation confirms their effectiveness across diverse population settings. Investigating deep learning algorithms also holds potential, although they typically require larger datasets and often lack interpretability [58]. Additionally, this study highlights the advantages and synergistic potential of combining different or multimodal spectroscopic techniques, offering useful analytical tools for assessing cartilage health and grading OA.

This review has several limitations. Firstly, the review includes only ten studies. This limited number can be attributed to our search strategy and inclusion criteria, which concentrated exclusively on the predictive performance of RS in OA assessment in human samples. Additionally, RS has not yet been widely accepted as a standard clinical diagnostic tool, resulting in a scarcity of available pre-clinical and clinical research. Secondly, the reference standards for confirming OA diagnosis varied across studies, with histopathological grading being the most common. This method is primarily intended for research rather than clinical use. Histopathology reveals only the sample’s origin and remains an imperfect reference standard with inherent biases. Moreover, the performance of ML models is constrained by the accuracy of the reference standard used for sample analysis. Therefore, current histological scoring systems might need refinement to more accurately measure and describe characteristics of OA.

Lastly, all included studies were rated as high risk, primarily due to small sample sizes, lack of external validation, and incomplete methodological reporting. However, to ensure consistent and transparent assessment, and to avoid publication bias, we applied a single validated framework, the PROBAST tool [34], and reported these studies to capture all available evidence along with their shortcomings [36]. Most studies exhibited pseudo-replication, where the number of samples exceeded the number of participants, and were predominantly based on ex vivo research. While such designs are common in preliminary investigations, large-scale studies with sufficient statistical power are essential to rigorously evaluate methodological robustness and reliability [56, 59]. ML models trained and tested on the same dataset often show high comparability but limited generalizability. To ensure real-world applicability, future work must prioritize applying trained models to new datasets and rigorously testing their generalization capacity. However, external validation requires additional samples from new donors, which can be challenging due to issues with donor participation, collaboration, and funding [56, 59]. Most included studies reported performance metrics such as accuracy, sensitivity, and specificity, which represent the predictive classification ability of the employed ML models. The consistency of these metrics, each addressing similar aspects of model accuracy and reliability, reflects a shared understanding of optimal evaluation methods [57]. Nonetheless, the clinical implementation of these ML models remains largely theoretical and has yet to be fully realized.

To more effectively establish the role of RS combined with ML models in OA assessment and to advance their application in clinical settings, future studies should involve a large sample size and a complete external validation setup, preferably using a longitudinal study design. Furthermore, using data from different populations to validate ML models can lead to discrepancies between the training data or environment and the validation environment, which may affect the ML models’ predictive accuracy [60]. Thus, documenting the characteristics of the health systems could underscore the differences between the data used for training and validating the models. However, integrating RS into diagnostic processes faces technical challenges like optimizing wavelengths and laser power, and standardizing data analysis parameters [56, 59]. Developing purpose-built cohorts for ML model analysis is essential to advance predictive modeling into effective, personalized clinical tools. Moreover, advancing in vivo research and refining ML analysis are critical for validating ML models’ clinical applicability and improving discrimination capabilities. Additionally, the feasibility and cost-effectiveness of implementing RS in clinical settings require further exploration.

## Conclusion

RS shows promising potential for accurately predicting and assessing the severity of OA, but the current evidence is still preliminary, as most studies rely on internal validation, have a high risk of bias, and lack external validation, which restricts the generalizability of these findings at this stage. To strengthen the evidence base for RS, future research should focus on developing and implementing standardized RS protocols to enable consistent comparisons across studies conducted in different clinical settings. Furthermore, ensuring the clinical applicability of these ML models requires external validation to reduce bias and prevent overfitting. Meanwhile, the predictive performance of RS must be rigorously investigated and standardized, aiming to integrate it as a routine clinical diagnostic tool in the foreseeable future.

## Supplementary Information

Below is the link to the electronic supplementary material.

Supplementary Material 1

## Data Availability

All data related to this study are available in this article.

## Abbreviations

*BRamS*: Brillouin and Raman micro-spectroscopy
*CRS*: Confocal Raman spectroscopy
*ICRS*: International Cartilage Repair Society
*K/L*: Kellgren-Lawrence
*LDA*: Linear discriminant analysis
*LOOCV*: Leave-one-out cross-validation
*LV*: Latent variable
*MIR*: Mid-infrared
*ML*: Machine learning
*MRI*: Magnetic resonance imaging
*NIR-RS*: Near-infrared Raman spectroscopy
*OA*: Osteoarthritis
*OARSI*: Osteoarthritis Research Society International
*PCA*: Principal component analysis
*PCs*: Principal components
*PLS-DA*: Partial least squares discriminant analysis
*PRISMA*: Preferred Reporting Items for Systematic Reviews and Meta-Analyses
*PROBAST*: Prediction Model Risk of Bias Assessment Tool
*PROSPERO*: International Prospective Register of Systematic Reviews
*PRS*: Polarized Raman spectroscopy
*RF*: Random forest
*ROB*: Risk of bias
*RS*: Raman spectroscopy
*RRS*: Resonance Raman spectroscopy
*SERS*: Surface-enhanced Raman scattering
*SVM*: Support vector machine
